# Transcriptome-wide association study uncovers the role of essential genes in anthracycline-induced cardiotoxicity

**DOI:** 10.1038/s41525-021-00199-4

**Published:** 2021-05-21

**Authors:** Erika N. Scott, Galen E. B. Wright, Britt I. Drögemöller, Jafar S. Hasbullah, Erandika P. Gunaretnam, Fudan Miao, Amit P. Bhavsar, Fei Shen, Bryan P. Schneider, Bruce C. Carleton, Colin J. D. Ross

**Affiliations:** 1 Faculty of Medicine, Department of Medical Genetics, University of British Columbia, Vancouver, BC Canada; 2 British Columbia Children’s Hospital Research Institute, Vancouver, BC Canada; 3 Faculty of Pharmaceutical Sciences, University of British Columbia, Vancouver, BC Canada; 4 Division of Translational Therapeutics, Department of Pediatrics, Faculty of Medicine, University of British Columbia, Vancouver, BC Canada; 5 Division of Hematology/Oncology, Department of Medicine, Indiana University, Indianapolis, IN USA; 6 Pharmaceutical Outcomes Programme, British Columbia Children’s Hospital, Vancouver, BC Canada; 7 Present Address: Faculty of Medicine & Dentistry, Department of Medical Microbiology & Immunology, University of Alberta, Edmonton, AB Canada

**Keywords:** Gene regulation, Pharmacogenomics, Transcriptomics, Gene expression, RNAi

## Abstract

Anthracyclines are highly effective chemotherapeutic agents; however, their clinical utility is limited by severe anthracycline-induced cardiotoxicity (ACT). Genome-wide association studies (GWAS) have uncovered several genetic variants associated with ACT, but the impact of these findings requires further elucidation. We conducted a transcriptome-wide association study (TWAS) using our previous GWAS summary statistics (*n* = 280 patients) to identify gene expression-related associations with ACT. We identified a genetic association between decreased expression of *GDF5* and ACT (*Z*-score = −4.30, *P* = 1.70 × 10^−5^), which was replicated in an independent cohort (*n* = 845 patients, *P* = 3.54 × 10^−3^). Additionally, cell viability of *GDF5*-silenced human cardiac myocytes was significantly decreased in response to anthracycline treatment. Subsequent gene set enrichment and pathway analyses of the TWAS data revealed that genes essential for survival, cardioprotection and response to anthracyclines, as well as genes involved in ribosomal, spliceosomal and cardiomyopathy pathways are important for the development of ACT.

## Introduction

Anthracyclines are prescribed for a wide range of cancers and are highly effective chemotherapeutic agents, contributing to the current >80% 5-year survival rate of childhood cancers^1^. The clinical utility of these chemotherapeutic agents, however, is limited by the debilitating adverse effect of anthracycline-induced cardiotoxicity (ACT). ACT most commonly presents as subclinical cardiac dysfunction but can progress to severe congestive heart failure in up to 16% of paediatric patients^2,3^.

Genome-wide association studies (GWAS) have identified variants associated with ACT^4–7^; however, the impact of some of these variant-level associations on ACT is still being explored. In addition, many GWAS hits reside in non-coding regions, further complicating the assignment of function to these variants. Recent years have seen a development in the understanding of non-coding regions, and, consequently, many variants occurring in these regions are now known to affect the regulation of neighbouring genes^8^. Expression quantitative trait loci (eQTLs)—genetic variants that influence expression of nearby genes—provide a link between non-coding variation and potential downstream functional consequences^9^.

Building on this knowledge, large publicly available datasets such as those generated by the Genotype-Tissue Expression (GTEx) Project, which contains eQTL data for a variety of tissues^9,10^, can be exploited to study gene expression differences in particular tissues of interest to further elucidate gene-level associations with a specific trait. Transcriptome-wide association studies (TWAS) have thus come to the forefront as a means of integrating transcriptomic and GWAS information and harnessing the power of these large-scale datasets^11^. Further, using Connectivity Map (CMap)^12,13^, these data can be leveraged for gene expression compound signature matching to guide the development of novel treatment strategies.

In pharmacogenomics, eQTLs have been shown to be highly associated with drug susceptibility phenotypes, highlighting the importance of studying eQTLs in this field of research^14^. In addition, recent RNA-seq studies have harnessed these resulting changes in gene expression to further investigate and uncover the genetic components of ACT^15–17^. To this end, we conducted a TWAS, combining GTEx eQTL information from diverse tissues^9,10^ with our previous GWAS summary statistics^4^, to identify gene expression-related associations with ACT and reveal potential mechanisms underlying the development of this adverse reaction. We subsequently performed replication and functional validation analyses on the top association to further investigate its role in the development of ACT. In addition, using the gene expression profiles generated from our TWAS, we utilised CMap to identify potential cardioprotectants for future mechanistic investigation in ACT.

## Results

**Fig. 1:**
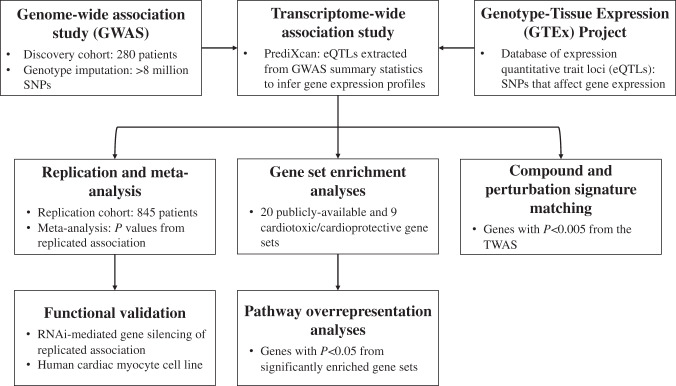
Overview of methods. A TWAS was initially conducted using both previous GWAS data and GTEx gene expression information to identify differentially expressed genes associated with ACT. From there, replication of the top associations in an independent cohort and a meta-analysis as well as subsequent functional validation were conducted. Gene set enrichment and pathway analyses identified gene lists and pathways overrepresented in the TWAS. Finally, compound and perturbation gene expression signature matching was conducted to identify compounds and perturbations with similar or dissimilar expression profiles to the TWAS genes.

A brief overview of the methods is presented in Fig. 1.

### Transcriptome-wide association study analyses

**Fig. 2:**
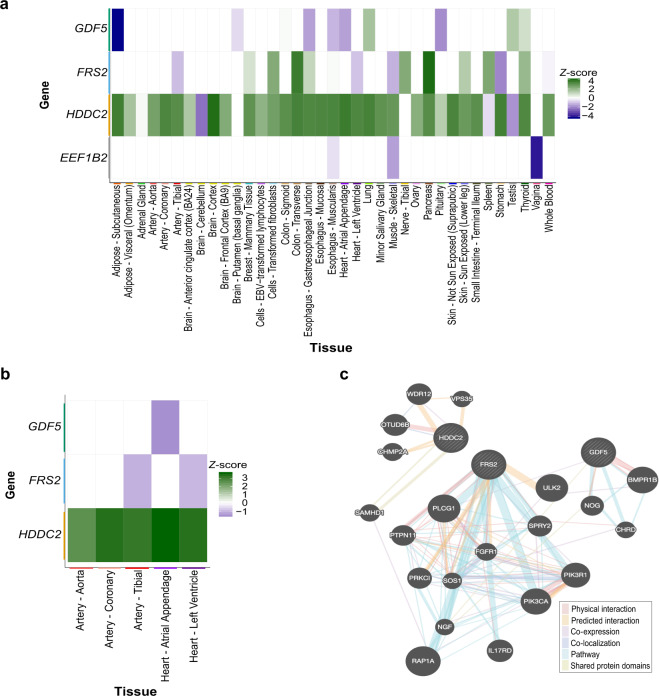
ACT TWAS identifies novel candidate genes based on expression profiles. **a** Analysis of diverse tissues identified four genes that passed an FDR correction for multiple testing in the TWAS and indicate that expression may modify ACT risk. Gene-level *Z*-scores, reflecting the number of standard deviations the mean expression of the gene (adjusted for the weight of each eQTL influencing expression of the gene) in ACT cases is from that of controls, across tissues where these genes were detected are plotted. **b** Association of top TWAS associated genes in tissues most relevant for ACT (i.e. heart and arterial). *Z*-score > 0 (green) indicates increased expression in ACT cases, while *Z*-score < 0 (purple) indicates decreased expression in these patients. **c** Gene interaction networks for the top TWAS associated genes identified using GeneMANIA.

To study heritable gene expression differences associated with ACT, we used summary level data from the discovery cohort of our previous GWAS^4^, and inferred gene expression profiles across all tissue types with S-PrediXcan^18^. Expression levels of four genes (*GDF5*, *FRS2, HDDC2* and *EEF1B2*) passed the Benjamini–Hochberg FDR < 0.2 threshold and were significantly associated with ACT across various tissues (Table 1 and Fig. 2a).

**Table 1:** Top genes across all tissues from the ACT TWAS.

Gene	*Z*-score^a^	*P* value	Tissue
*GDF5*	−4.30	1.70 × 10^−5^	Adipose - subcutaneous
*FRS2*	4.07	4.67 × 10^−5^	Pancreas
*HDDC2*	4.01	6.08 × 10^−5^	Brain - cortex
*EEF1B2*	−3.97	7.24 × 10^−5^	Vagina

^a^Represents the number of standard deviations the mean expression of the gene in ACT cases is from that of controls, after adjusting for the weight of each eQTL influencing expression of the gene.

Since ACT occurs in cardiac tissue, we specifically examined heart and arterial tissues to investigate the predicted heritable expression of the top genes in these tissues. None of the four genes associated with ACT passed the Benjamini–Hochberg FDR < 0.2 threshold for statistically significant differential expression in heart and arterial tissues, but we nevertheless examined non-significant expression in these tissues as a discovery analysis. *GDF5* expression was lower in ACT cases in the atrial appendage of the heart (*Z*-score = −1.53), while *FRS2* expression was also decreased in cases in both the tibial artery (*Z*-score = −1.07) and left ventricle of the heart (*Z*-sore = −0.96; Fig. 2b and Supplemetary Table 1). Increased expression of *HDDC2* in ACT cases was detected across all heart and arterial tissues, with the most significant association in the atrial appendage (*Z*-score = 3.61, *P* = 3.04 × 10^−4^; Fig. 2b and Supplementary Table 1). *EEF1B2* was not differentially expressed in heart or arterial tissue in the TWAS and thus was excluded from further analyses (Supplementary Table 1). Examination of the three genes that were expressed in heart and/or arterial tissues using GeneMANIA^19^ revealed many known and predicted interactions among *GDF5*, *FRS2* and *HDDC2* with other genes (Fig. 2c).

### Replication of the top TWAS associations in an independent cohort

Summary statistics were obtained from another ACT GWAS in adult patients of European American ancestry with breast cancer conducted by Schneider et al.^5^ (*n* = 845 patients), and a TWAS was performed to investigate across-tissue associations of the three prioritised genes in an independent cohort. Only the *GDF5* association replicated in this cohort (top tissue *Z*-score = −2.62, *P* = 3.54 × 10^−3^; Table 2). In addition, we conducted a meta-analysis on the *P* values of the *GDF5* association in our cohort and that of Schneider et al.^5^, and the *GDF5* association remained significant (*P* = 3.69 × 10^−5^; Table 2).

**Table 2:** Replication and meta-analysis of the *GDF5* association.

Cohort	Top tissue *Z*-score	*P* value^a^
This study	−4.30	7.53 × 10^−4^
Schneider et al.	−2.62	3.54 × 10^−3^
Meta-analysis	–	3.69 × 10^−5^

^a^Derived from S-MultiXcan analyses.

### Functional validation of *GDF5* in human cardiac myocytes

**Fig. 3:**
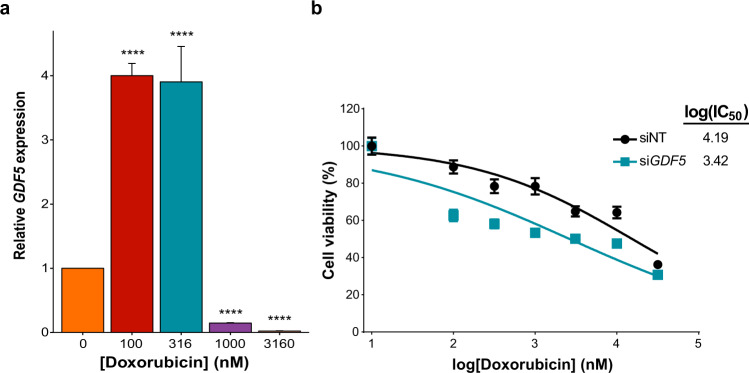
In response to increasing concentrations of doxorubicin, *GDF5* undergoes gene expression changes and alters HCM cell viability when silenced. **a** Expression levels of *GDF5* at increasing concentrations of doxorubicin were measured in HCM cells, normalised to the housekeeping gene *HPRT1*, and compared using one-way ANOVA to baseline (0 nM doxorubicin). Two independent experiments were performed (total *n* = 6) and their results combined. ****P < 0.0001 after Bonferroni multiple testing correction. Data are presented as mean and standard deviation. **b** Expression levels of *GDF5* were silenced with RNAi and HCM cell viability at increasing concentrations of doxorubicin was measured using an MTT assay (*n* = 5). Dose-response curves were fitted to a non-linear regression, log(inhibitor) versus normalised response 4 parameter model and data are presented as mean and standard deviation. siNT represents negative control non-targeting siRNA. The logarithm of the half maximal inhibitory concentrations (IC_50_) are shown.

Given that the association between decreased *GDF5* expression and ACT was observed in two independent cohorts, we conducted functional validation experiments to further examine this association in vitro. Human cardiac myocyte (HCM) cells were exposed to increasing levels of doxorubicin and *GDF5* expression was assessed. Gene expression of *GDF5* was significantly increased at lower concentrations of doxorubicin and significantly decreased at higher concentrations of doxorubicin (*P* < 0.0001; Fig. 3a).

Since the expression levels of *GDF5* were downregulated in heart and arterial tissues in the TWAS cases, we investigated whether silencing *GDF5* affected HCM cell viability in vitro. HCM cells with silenced *GDF5* (si*GDF5*) showed enhanced susceptibility to doxorubicin compared with non-targeting control (siNT) and a significantly different log(IC_50_) from that of siNT (*P* < 0.0001; Fig. 3b).

### Gene set enrichment analyses

To determine what types of genes were enriched for association with ACT in the TWAS, gene set enrichment analyses were performed using: (1) gene sets derived from publicly-available respositories of genes grouped by family or function and (2) gene sets derived from RNA-seq data collected after treating cells with either cardiotoxic (doxorubicin)^16^ or cardioprotective (ATRA) agents (Supplementary Table 2). Examination of the publicly-available gene sets revealed that gene sets essential in mice, essential in culture, and intolerant to loss of function mutations in humans were significantly enriched across all tissues in ACT cases after correcting for multiple testing (Table 3). Only the essential in culture gene set remained significantly enriched in heart and arterial tissues after correcting for multiple testing (Supplementary Table 3).

**Table 3:** Significantly enriched gene sets across all tissues in the TWAS of ACT.

Gene set	Mean *Z*^2^ (gene set)	Mean *Z*^2^ (all genes)	*P* value^a^
Publicly-available gene sets			
Essential in mice	0.86	0.82	2.00 × 10^−7^
Essential in culture	0.96	0.82	0.04
Human LoF intolerant	0.86	0.81	1.36 × 10^−5^
Cardiotoxic/cardioprotective gene sets			
ATRA (downregulated)	0.90	0.82	1.35 × 10^−3^
Cluster 5^b^	0.87	0.82	3.60 × 10^−4^

*LoF* loss of function, *ATRA* all-trans retinoic acid.

^a^Bonferroni adjusted.

^b^Described by Knowles et al.^16^

These analyses reveal the importance of essential genes in mice, culture, and humans, as well as those related to cardiotoxic/cardioprotective agents.

Examination of the cardiotoxic/cardioprotective gene sets revealed that: (1) genes that were downregulated upon treatment with ATRA, and (2) genes upregulated only at lower doses of anthracyclines (i.e. cluster 5)^16^ were significantly enriched in the TWAS data across all tissues after correcting for multiple testing (Table 3 and Supplementary Table 2).

### Pathway overrepresentation analyses

To further investigate underlying biological mechanisms and pathways, we extracted nominally significantly differentially expressed genes (*P* < 0.05) from the enriched gene sets. Pathways with an enrichment ratio (ER) > 10 were prioritised, indicating that the number of genes observed in that particular pathway was ten-fold greater than expected. Significantly overrepresented pathways with ER > 10 included ribosome (ER = 24.72, *P* = 3.17 × 10^−11^), spliceosome (ER = 22.2, *P* = 1.46 × 10^−10^), hypertrophic cardiomyopathy (ER = 12.31, *P* = 1.15 × 10^−3^), dilated cardiomyopathy (ER = 11.50, *P* = 4.93 × 10^−3^), and arrhythmogenic right ventricular cardiomyopathy (ER = 11.35, *P* = 1.23 × 10^−3^; Table 4).

**Table 4:** Significantly overrepresented pathways with enrichment ratios > 10.

Pathway	Number of genes in pathway	Number of enriched genes	Expected number of genes	Enrichment ratio	*P* value	Enriched genes	Significantly enriched gene set
Ribosome	135	11	0.44	24.72	3.17 × 10^−11^	*RPL5*, *RPL11*, *RPL13*, *RPL23A*, *RPL32*, *RPL38*, *RPS5*, *RPS7*, *RPS9*, *RPS20*, *RPS28*	Essential in culture
Spliceosome	133	11	0.50	22.20	1.46 × 10^−10^	*SF3A1*, *SNRNP200*, *SF3B1*, *PRPF31*, *PRPF19*, *HNRNPC*, *HNRNPU*, *XAB2*, *SRSF1*, *SRSF3*, *PRPF3*	Essential in mice, essential in culture, human LoF intolerant
Hypertrophic cardiomyopathy	83	6	0.49	12.31	1.15 × 10^−3^	*ITGA3*, *ITGA7*, *TNNT2*, *TPM3*, *CACNA1S*, *ITGA8*	Essential in mice, ATRA (downregulated)
Arrhythmogenic right ventricular cardiomyopathy	74	5	0.43	11.50	4.93 × 10^−3^	*ITGA3*, *ITGA7*, *PKP2*, *CACNA1S*, *ITGA8*	Essential in mice, ATRA (downregulated)
Dilated cardiomyopathy	90	6	0.53	11.35	1.23 × 10^−3^	*ITGA3*, *ITGA7*, *TNNT2*, *TPM3*, *CACNA1S*, *ITGA8*	Essential in mice, ATRA (downregulated)

*LoF* loss of function, *ATRA* all-trans retinoic acid.

These analyses identify ribosome, spliceosome and cardiomyopathy pathways as important for the development of ACT.

### Compound and perturbation gene expression signature matching

**Fig. 4:**
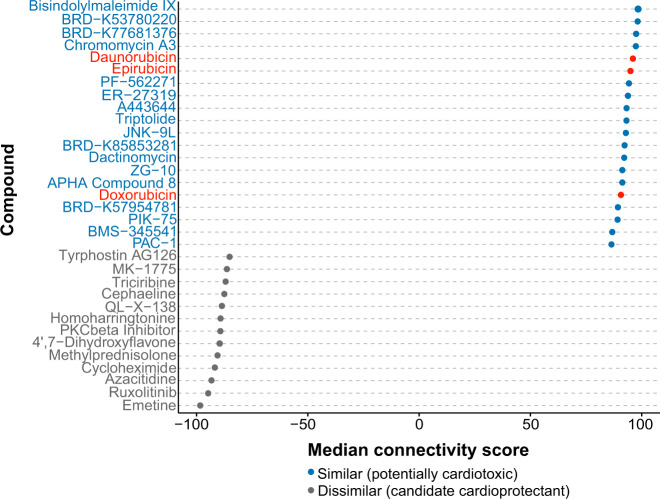
Compounds with highly similar and dissimilar gene expression signatures to the TWAS. Expression signatures of compounds in CMap were compared with those of the TWAS. Compounds with a median connectivity score > 90 (similar gene signatures) or < −90 (dissimilar gene signatures) are shown. Anthracyclines are highlighted in red.

We used CMap^12,13^ to compare gene expression signatures of the TWAS with those of 19,811 different small molecule compounds to identify compounds with similar and dissimilar expression signatures to those of the TWAS for prioritisation of cardioprotective agents for future functional studies. Since TWAS gene expression signatures based on *Z*-scores reflect ACT cases, compounds with similar signatures could represent potential cardiotoxic agents while compounds with dissimilar signatures could represent potential cardioprotective agents. Among the compounds with similar gene expression signatures are the anthracyclines doxorubicin, daunorubicin and epirubicin (Fig. 4).

In addition, we compared gene expression signatures of the TWAS with those generated after a perturbation (gene knockdown) to investigate genes that, when knocked down, produce similar or dissimilar gene expression profiles to the ACT cases. Three genes known to be involved in splicing were identified; two of these genes (*UBL5* and *PRPF4*) produced highly dissimilar expression profiles to the ACT cases when knocked down, while *PUF60* produced similar gene expression profiles when knocked down (Supplementary Fig. 1).

## Discussion

Anthracyclines are widely used in the clinic due to their anti-cancer effectiveness, but their utility is limited by ACT. Previous GWAS^4–7^ and transcriptomic analyses of doxorubicin-treated human pluripotent stem cell-derived cardiomyocytes (hPSC-CMs)^15–17^ have uncovered genetic associations with ACT. Although these approaches have yielded important insights into the biology underlying ACT, a substantial proportion of the heritability associated with this adverse event remains unaccounted for. Therefore, we performed a TWAS of ACT to identify heritable expression profiles associated with the development of this adverse drug reaction in an integrative manner.

TWAS analyses revealed that *GDF5* (growth differentiation factor 5) was downregulated across many tissues in ACT cases compared with controls (Fig. 2a and Supplementary Table 1).

The *GDF5* association was then replicated in an independent cohort^5^ (Table 2). To ensure that the results derived from these TWAS analyses are robust and transferrable across populations, replication was performed in a completely independent population. However, we recognise that, because the discovery and replication studies were conducted in cohorts of different ages, there are differences between them such as in the cumulative dose of anthracyclines used and assessment of cardiotoxicity. Therefore, future studies aimed at replication in clinically similar cohorts may uncover additional factors that are unique to paediatric or adult oncology patients.

Silencing *GDF5* in HCM cells in vitro resulted in significantly decreased cell viability after treatment with various concentrations of doxorubicin, providing further evidence for the role of *GDF5* in ACT (Fig. 3b). Since decreased expression of *GDF5* is associated with increased risk of ACT, these results support the involvement of *GDF5* in protective mechanisms in cardiomyocytes against cardiotoxic agents at clinically relevant doses.

In non-cardiac tissue, GDF5 is a bone morphogenetic protein and member of the TGF-β superfamily that has long been known to be involved in bone and cartilage formation during development^20^. Genetic variation within this gene has thus been associated with bone-related traits such as differences in human height^21^, hip dysplasia^22^, and knee pain^23^. In cardiac tissue, TGF-β signalling cascades play roles in cardiac repair and remodelling^24^. Indeed, *GDF5* was previously linked to cardiac repair after myocardial infarction in mice^25^. Zaidi et al.^25^ showed that *Gdf5*-knockout mice displayed incomplete cardiac remodelling after induced myocardial infarction, as well as increased dilation of the left ventricle, increased fibrosis and apoptosis, and decreased abundance of vessels. Given the close proximity of the atrial appendage to the left ventricle and the fact that removal of the left atrial appendage leads to significant changes in the left ventricle, as well as the relationship between dysfunction of the left ventricle and atrial appendage in heart disorders such as atrial fibrillation, congestive heart failure, and myocardial infarction^26,27^, it is conceivable that pre-existing dysregulation of an important gene such as *GDF5* in the atrial appendage may affect left ventricle function. As most studies examining the role of *GDF5* in cardiac repair were in the context of myocardial infarction, future functional studies to determine the exact role that downregulation of *GDF5* plays in the context of global transcriptional toxicity (e.g. assessed via RNA-seq in patient-derived cells) are warranted.

To further investigate the role of specific groups of genes in ACT, enrichment analyses were performed. These analyses revealed that the three significantly enriched gene sets that were derived from publicly-available data all fall under the common theme of genes that are essential for survival (essential in mice, essential in culture, and human loss of function intolerant; Table 3). This indicates that genetic variants that alter expression of such genes modify ACT risk, but do not completely abolish the function. Interestingly, *GDF5* was found within the human loss of function intolerant gene set, further highlighting the importance of this gene and concordance with the enrichment analyses.

Pathway overrepresentation analyses, to further investigate which differentially expressed genes were driving the significant enrichments in these gene sets, identified five pathways with ERs > 10 (Table 4). The top pathway, the ribosome (Table 4), was supported by Holmgren et al.^28^ who also identified overrepresentation of the ribosome pathway in their analyses of differential protein expression among hPSC-CMs treated with doxorubicin. They also showed that although genes involved in apoptotic signalling were differentially expressed upon immediate treatment with doxorubicin, shifts in metabolism and energy conservation become more important over time and ribosomal genes become increasingly downregulated^28^. Taken together, these observations suggest a role for ribosomal genes in conferring susceptibility to ACT by affecting the mechanism by which cardiomyocytes respond to doxorubicin treatment.

The second significantly overrepresented pathway, the spliceosome (Table 4), was also supported by prior studies. Knowles et al.^16^ discovered that in hPSC-CMs treated with various doxorubicin concentrations, doxorubicin exposure led to the increased use of cryptic splice sites and resulted in reduced splicing fidelity in many genes. In addition, CMap gene knockdowns resulting in highly similar and dissimilar expression signatures to those of the TWAS included three genes known to play roles in splicing: *PUF60*, *PRPF4* and *UBL5* (Supplementary Fig. 1)^29–31^. Variants changing the expression of genes involved in splicing may, therefore, predispose an individual to ACT by further exacerbating the effect of doxorubicin on splicing fidelity.

The three cardiomyopathy pathways that were overrepresented in our dataset were also overrepresented in the Holmgren et al.^28^ study (Table 4). Thus, susceptibility to other cardiomyopathies may predispose certain individuals to ACT when receiving doxorubicin.

Examination of cardiotoxic/cardioprotective gene sets revealed an enrichment of genes whose expression is (1) decreased upon treatment of HCM cells with the cardioprotective agent ATRA, and (2) increased upon treatment with lower (and more clinically relevant) concentrations of the cardiotoxic agent doxorubicin. Similar to the publicly-available gene sets, the difference in mean *Z*^2^ in general increased as the tissues became more specific to ACT (Table 3 and Supplementary Table 3), but associations for heart and arterial tissues were not significant after multiple testing correction (Supplementary Table 3), suggesting a need for larger cohorts.

With regards to the doxorubicin gene set, Knowles et al.^16^ treated hPSC-CMs from 45 individuals with various concentrations of doxorubicin (0.625–5 µM) and conducted RNA-seq to identify unique doxorubicin response gene clusters across the gradient of concentrations. The authors identified six different clusters, from which cluster 5 was significantly enriched in our dataset^16^. This cluster contained genes that were initially upregulated at lower concentrations of doxorubicin and then gradually downregulated at higher concentrations^16^. Knowles et al.^16^ found an overrepresentation of genes relating to targets of p53 in this cluster, suggesting that this cluster is related to DNA damage response. Similarly, Reyes et al.^17^ treated hPSC-CMs with various doxorubicin concentrations and identified a set of significantly downregulated genes with roles in DNA damage repair connected to p53. These findings suggest that the DNA damage response pathway may play an important role in the development of ACT. In our functional analyses, *GDF5* exhibited the same pattern of upregulation at lower concentrations and downregulation at higher concentrations of doxorubicin, suggesting overlapping response mechanisms to genes within cluster 5.

Pathway overrepresentation analyses, to further investigate which genes were driving the significant enrichments in these gene sets, identified the same three cardiomyopathies that were identified in the pathway overrepresentation analyses for the significantly enriched publicly-available gene sets (Table 4).

Anthracyclines such as doxorubicin, daunorubicin and epirubicin are distributed among the compounds with similar gene expression signatures to the ACT-associated gene expression profiles that were derived from the TWAS analyses (Fig. 4). As these chemotherapeutic agents are known to be cardiotoxic, these results provide further support for the role that the heritable gene expression profiles play in ACT.

Compounds with highly dissimilar expression signatures to those of ACT cases were of interest because they could potentially be explored as cardioprotectants for ACT. As such, these compounds were further investigated for any role they might play in cardioprotection. While dexrazoxane, one of the most well-known cardioprotectants for ACT, is not found in the CMap database, and thus is not on our list of potential cardioprotectants, other compounds show promise as potential cardioprotectants. Of note, methylprednisolone, a glucocorticoid receptor agonist and anti-inflammatory agent, has some evidence for a cardioprotective role^32,33^. Although it is known that glucocorticoid signalling is important for normal cardiac function, such that mice lacking the glucocorticoid receptor die of heart failure^34^, the role that glucocorticoid signalling plays in ACT has yet to be elucidated. Interestingly, a recent study in mice determined that activating the glucocorticoid receptor along with antagonising the mineralocorticoid receptor in cardiomyocytes may be cardioprotective and useful for treating heart failure^35^. Given that methylprednisolone is a glucocorticoid receptor agonist with minimal mineralocorticoid receptor binding^36^, it could be a potential candidate for future functional studies. In addition, a recent GWAS in anthracycline-treated cancer patients identified a genetic variant potentially located in the binding site of a glucocorticoid receptor as highly associated with ACT^5^. This variant is predicted to reduce glucocorticoid receptor binding and affect downstream signalling, possibly uncovering the importance of glucocorticoid signalling in ACT and cardioprotection. As our analyses were mainly to prioritise potential cardioprotective compounds, future functional studies in ACT cellular or animal models are warranted.

We appreciate our study was not without limitations. First, our discovery cohort was relatively small (*n* = 280 patients). This reflects the rarity of paediatric cancer and subsequent identification of adverse drug reactions in these patients, but also highlights the need for the development of collaborative networks to gather large cohorts of uniformly-treated patients. Second, although we performed fine-mapping of the top TWAS hits using FOCUS^37^, the results indicated that our study was underpowered for these analyses. To address these two power-related limitations, we conducted replication analyses in an independent cohort and functional analyses in HCM cells. Finally, a current limitation with the CMap database is that often the gene signature of only one dose of the compound is reported, such that gene signatures produced by other, potentially more therapeutically relevant doses, may be unknown. For example, ATRA only has a gene signature derived from a dose of 10 µM in the CMap database. This is much larger than the more therapeutically-relevant dose of 250 nM^38^ used in our analyses, likely explaining why ATRA is also absent from our list of potential cardioprotectants. It is therefore important for future studies to test the correct therapeutic doses of these potential cardioprotectants in appropriate cellular and animal models to determine whether they are truly cardioprotective and that they do not affect the efficacy of anthracycline therapy.

In conclusion, we conducted transcriptome-level analyses to find functionally-relevant genetic associations with ACT using a gene-based method, rather than a genetic variant-based method, to enable identification of genetic associations. Although our discovery cohort was relatively small (*n* = 280 patients), our top association with ACT (downregulation of *GDF5*) replicated in a larger independent cohort (*n* = 845 patients), and was functionally validated through gene silencing in HCM cells. We also determined that genes essential for survival, cardioprotection, and response to doxorubicin are important for the development of, or susceptibility to, ACT. In addition, we show that pathways involved in the ribosome, spliceosome, and other cardiomyopathies are predicted to be dysregulated in patients who have developed ACT and thus variants in these genes may predispose certain patients receiving anthracycline treatment to develop ACT. Finally, we have identified compounds with highly dissimilar gene expression signatures to those of the TWAS that represent candidate cardioprotectants for future functional studies. This study has therefore contributed significantly to improving our understanding of the genetics of ACT and has important implications for future strategies to reduce the burden of this adverse drug reaction.

## Methods

### Transcriptome-wide association study analyses

GWAS data were obtained from the European discovery cohort described in a previous publication^4^ and in Supplementary Table 4. Genotype phasing and imputation were performed using SHAPEIT2^39^ and IMPUTE2^40^, respectively. After quality control, 272 samples were available for analysis. Logistic regression was then performed using SVS v8.8.1 (Golden Helix, Bozeman, MT, USA) under an additive model, adjusting for age at start of treatment, cumulative anthracycline dose, tumour type (acute lymphoblastic leukaemia, Ewing’s sarcoma, and rhabdomyosarcoma) and cardiac radiation therapy. These data were used in conjunction with prediction models generated from the v7 release of the GTEx data for all available tissues (*n* = 48; downloaded from the PredictDB Data Repository, http://predictdb.org/, on 14 December 2017) to impute gene expression profiles and perform a TWAS using the September 2018 version of S-PrediXcan (single tissue associations) and October 2019 version of S-MultiXcan (across tissue associations), as previously described^18,41^, in order to identify differentially expressed genes associated with ACT. *Z*-scores were used to describe the differential expression of genes and represent the number of standard deviations the mean expression of a particular gene in ACT cases is from that of controls, after adjusting for the weight of each eQTL that influences the expression of the gene^18^. *Z*-scores < −1.96 and > 1.96 correspond to *P* < 0.05.

Human protein-coding genes (GRCh37) were isolated using the biomaRt^42,43^ package in R (v3.5.1) and these genes were included in all downstream analyses. Genes across all tissues in the S-PrediXcan^18^ analyses were considered to be statistically differentially expressed between cases and controls if they passed a Benjamini-Hochberg false discovery rate (FDR) < 0.2 threshold within a particular tissue. Heatmaps of TWAS *Z*-scores across tissues for genes significant after correction for multiple testing were generated using the ggplot2^44^ package in R. GeneMANIA^19^ was used to visualise interaction networks for the top differentially expressed genes. Fine-mapping to narrow down the causal region was performed using FOCUS^37^.

### Replication of the top TWAS associations in an independent cohort

Additional summary statistics were obtained from Schneider et al.^5^ and TWAS analyses were performed using S-MultiXcan^41^ for replication purposes. A replication *P* < 0.017 (*n* = 3 genes) threshold was used to identify significant associations. A meta-analysis of S-MultiXcan *P* values using Fisher’s method was then conducted with the metap^45^ package in R. Supplementary Table 4 provides a comparison of the discovery and replication cohorts and corresponding GWAS analyses.

### Functional validation of *GDF5* in human cardiac myocytes

Human cardiac myocyte (HCM) cells (Cat# C-12810), isolated from ventricular tissue of the adult human heart were purchased from PromoCell (Heidelberg, Germany) and cultured in Myocyte Growth Medium supplemented with SupplementMix containing epidermal growth factor, basic fibroblast growth factor, and insulin (PromoCell). Cultures were maintained at 37 °C and 5% CO_2_, and subcultured using DetachKit (PromoCell) according to manufacturer’s instructions. All experiments were conducted within passages 3 to 9.

In all, 2 × 10^5^ HCM cells were seeded into each well of a six-well plate in 2 mL growth medium and cultured overnight. The next day, fresh medium containing DMSO (vehicle control) or doxorubicin (100 nM, 316 nM, 1000 nM or 3160 nM) was added to cells. Concentrations were selected based on therapeutic concentrations of doxorubicin^46,47^. Cells were grown for 24 h, total RNA was extracted using the PureLink™ RNA Mini Kit (Thermo Fisher Scientific, Waltham, MA, USA), and cDNA was synthesised using 500 ng extracted RNA. Subsequent quantitative PCR (qPCR) was conducted in a 10 µL reaction volume using 5 µL 2x TaqMan Universal Master Mix, 0.5 µL TaqMan probe, and 2 µL cDNA on the QuantStudio™ 7 Flex Real-Time PCR System (Thermo Fisher Scientific) under standard cycling conditions with validated TaqMan gene expression assay probes Hs01003267_m1 (*HPRT1*) and Hs00167060_m1 (*GDF5*). *HPRT1*, a housekeeping gene whose expression levels remain constant across doxorubicin concentrations, was used as a control. Two independent experiments were performed (total *n* = 6 replicates) and their results combined.

Expression levels of *GDF5* were normalised to *HPRT1* as the reference gene and DMSO-treated cells as the calibrator, and relative expression levels were calculated using the 2^-∆∆Ct^ method. Mean relative expression levels at each doxorubicin concentration were compared to mean expression levels of DMSO-treated cells (0 nM doxorubicin) using one-way ANOVA and *P* values were adjusted for multiple testing (*n* = 4) using Bonferroni correction. Figures were plotted using the ggplot2^44^ and ggpubr^48^ packages in R.

Gene expression levels of *GDF5* were silenced in HCM cells using 25 nM siGENOME human SMARTpool siRNA; negative control wells were transfected with siGENOME Non-Targeting Control siRNA Pool #2 (Dharmacon, Lafayette, CO, USA).

4 × 10^3^ HCM cells were seeded into each well of a 96-well plate in 100 µL growth medium and cultured overnight. The next day, *GDF5* was silenced as described above, and siRNA transfection was performed using DharmaFECT 1 transfection reagent according to the DharmaFECT transfection protocol (Dharmacon). Gene expression was quantified by qPCR on the PikoReal Real-Time PCR System (Thermo Fisher Scientific) with standard cycling conditions and the TaqMan probes for *HPRT1* and *GDF5*. *HPRT1* expression levels were used as a positive control to ensure silencing was specific to *GDF5*. Under these conditions, >75% gene silencing was achieved (Supplementary Fig. 2A).

After 24 h, cells were treated with serial concentrations of doxorubicin (0, 100, 316 nM, 1, 3.16, 10, and 31.6 µM). Cell viability was assessed using an MTT assay 48 h after treatment with doxorubicin. Cells were incubated with MTT (MilliporeSigma, Burlington, MA, USA) for 3.5 h at 37 °C. After incubation, the supernatant was aspirated and each well was treated with DMSO for 15 min at room temperature. Absorbance was measured on a POLARstar Omega plate reader (BMG Labtech, Ortenberg, Germany) at an optical density of 590 nm. Cell viability in cells not treated with doxorubicin was assessed through absorbance measurements to ensure silencing alone did not cause cell death (Supplementary Fig. 2B). Five independent experiments were performed and their results combined.

Percent cell viability was normalised to untreated wells and mean and standard deviation were calculated for each concentration of doxorubicin. Dose-response curves were fitted to a non-linear regression, log(inhibitor) versus normalised response 4 parameter model and plotted in Prism v7 (GraphPad, La Jolla, CA, USA). The logarithm of the half maximal inhibitory concentrations (IC_50_) were compared using an extra-sum-of-squares F test.

### Datasets for gene set enrichment analyses

Diverse gene sets (Supplementary Table 2) were obtained from three different sources:

1. Publicly available repositories: diverse gene lists from both the MacArthur Lab repository, containing genes grouped by similar protein function or family (https://github.com/macarthur-lab/gene_lists, downloaded September 2018), and the Exome Aggregation Consortium (ExAC)^49^, containing genes that are intolerant to loss of function mutations in humans;
2. Six gene clusters that showed differential response to varying concentrations of doxorubicin in human pluripotent stem cell-induced cardiomyocytes (hPSC-CMs) identified by Knowles et al.^16^;
3. Genes differentially expressed upon treatment of rat heart (H9c2) cells with the cardioprotectant all-trans retinoic acid (ATRA), as described below in further detail.

For subsequent discussion, we grouped these gene sets into two categories: (1) publicly-available gene sets (MacArthur Lab and ExAC gene sets), and (2) cardiotoxic/cardioprotective gene sets (six anthracycline-induced gene clusters and genes that were differentially expressed upon treatment with ATRA).

To identify genes that were differentially expressed upon treatment with ATRA, H9c2(2-1) (ATCC Cat# CRL-1446, RRID: CVCL_0286) cells were purchased in December 2013 from the American Type Culture Collection (Cedarlane, Burlington, ON, Canada) and cultured in growth medium comprised of HyClone DMEM supplemented with 10% FBS, 1% l-glutamine, and 2% sodium bicarbonate. *Mycoplasma* testing was conducted in August 2015 by PCR of the cell culture media using positive control samples as reference. The number of passages between thawing and use in the below experiments (conducted in February 2016) was 4–25, and cells were maintained in a humidified incubator at 37 °C and 5% CO_2_. ATRA was purchased from MilliporeSigma and dissolved in DMSO according to manufacturer’s instructions.

H9c2 cells (200,000 cells/well) were plated into each well of a six-well dish in 2 mL growth medium. The following day, cells were treated with fresh growth medium containing either 250 nM ATRA or DMSO (vehicle control). After 24 h of treatment, cells were harvested and total RNA was immediately purified using the PureLink RNA Mini Kit with the PureLink DNase Set according to the manufacturer’s specifications (Thermo Fisher Scientific). RNA-seq was then performed on the NextSeq 500 (Illumina, San Diego, CA, USA) sequencing platform using TruSeq RNA sample prep and paired-end sequencing (2 × 75 bp). Data analyses were implemented by mapping FASTQ files to the Ensembl *Rattus norvegicus* reference transcriptome (Rnor_5.0) using the k-mer aligner, kallisto 0.44.0^50^. Differential expression was then subsequently determined using the sleuth^51^ package in R.

### Gene set enrichment analyses

To determine whether genes in a particular gene set were more likely to be up- or downregulated in the TWAS data compared with all other genes, a Wilcoxon rank sum test was used to compare mean *Z*^2^ statistics from each gene set with those of all other genes in the TWAS data as previously described^18^. *P* values were adjusted for the number of gene sets tested (20 publicly-available gene sets; 9 cardiotoxic/cardioprotective gene sets) using Bonferroni multiple testing correction to identify significantly enriched gene sets *(P* < 2.50 × 10^−3^ and *P* < 5.56 × 10^−3^, respectively). All analyses were performed across: (1) all tissues, (2) heart and arterial tissues only, and (3) heart tissues only.

### Pathway overrepresentation analyses

Significantly enriched gene sets were further interrogated by extracting genes within these gene sets that exhibited nominally significant associations (*P* < 0.05) in the TWAS analyses (up to a maximum of 150 genes). These genes were subsequently investigated using WebGestalt Overrepresentation Enrichment Analysis^52–54^ for overrepresentation in biological pathways. Each significantly enriched gene set was separated into three groups: upregulated genes (*Z*-score > 1.96), downregulated genes (*Z*-score < −1.96), and all genes (*Z*-score > 1.96 and < −1.96). Significantly enriched gene sets were also cross-compared with each other in pairs and the top overlapping genes with *P* < 0.05 were investigated using WebGestalt. Pathways that passed a Benjamini-Hochberg FDR < 0.05 were considered significant and those with an enrichment ratio (ER) > 10 were prioritised.

### Compound and perturbation gene expression signature matching

Genes with *P* < 0.005 in the TWAS were extracted and separated into up- (*Z*-score > 2.81) and down- (*Z*-score < −2.81) regulated groups. CMap^12,13^ was leveraged to identify compounds and perturbations (gene knockdowns) that produced highly similar or dissimilar gene expression signatures to the ACT profile (20 upregulated and 29 downregulated genes) derived from the TWAS for heart and arterial tissues only. Compounds and perturbations with median connectivity scores > 90 (similar gene signatures) or <−90 (dissimilar gene signatures) were prioritised and plotted using the ggplot2^44^ package in R.

### Reporting summary

Further information on research design is available in the Nature Research Reporting Summary linked to this article.

## Supplementary information

Supplemental Material

Reporting Summary

## Data Availability

The summary-level data analysed during this study are available through Dryad Digital Repository with the identifier doi:10.5061/dryad.k0p2ngf6j. The raw RNA-sequencing data from ATRA treatment of H9c2 cells has been deposited in the Sequence Read Archive under the accession number PRJNA717786.

## Code availability

No custom code was used in this study. Open source software including S-PrediXcan (September 2018), S-MultiXcan (October 2019), FOCUS (January 2021), and R (v3.5.1) were used to conduct the TWAS and subsequent fine-mapping, as well as in data analysis and visualisation. Other open source software such as GeneMANIA (September 2018), WebGestalt (September 2018) and CMap (September 2018), were used to visualise gene interaction networks, conduct pathway analyses, and identify candidate compounds for further analyses, respectively. SVS (v8.8.1) was used to perform logistic regression analyses and Prism (v7) was used to analyse the cell viability data and plot dose-response curves.
