# Supplementary material for: Transcriptome-wide association study uncovers the role of essential genes in anthracycline-induced cardiotoxicity
Source: NPJ Genom Med. 2021 May 21;6:35. doi: 10.1038/s41525-021-00199-4 (PMC8140137; doi:10.1038/s41525-021-00199-4)
Supplement: Supplementary file 1 — Supplemental Material [file 41525_2021_199_MOESM1_ESM.pdf]

Supplementary Figures

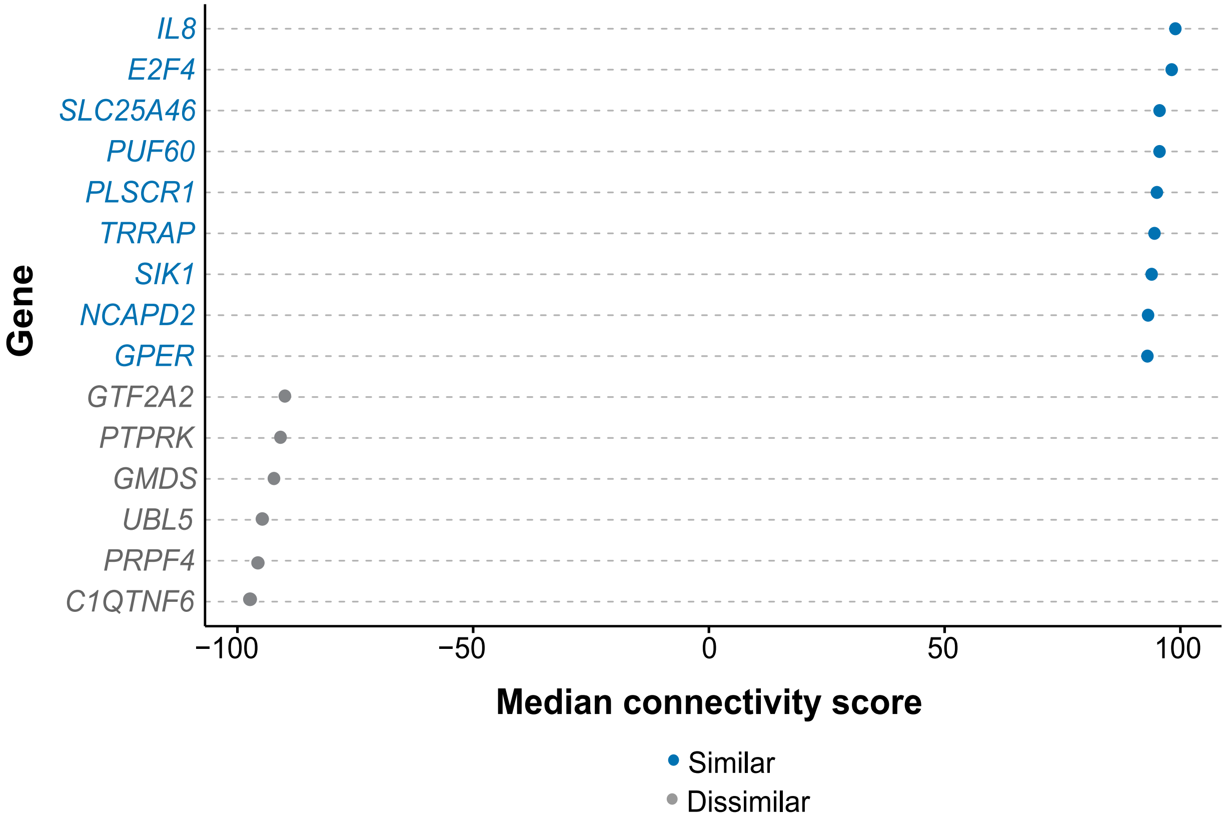

Supplementary Figure 1. Gene perturbations with highly similar and dissimilar gene expression signatures to cases in the TWAS. Expression signatures of perturbations (gene knockdowns) in CMap were compared with those of the TWAS. Perturbations with a median connectivity score > 90 (similar gene signatures) or < -90 (dissimilar gene signatures) are shown.

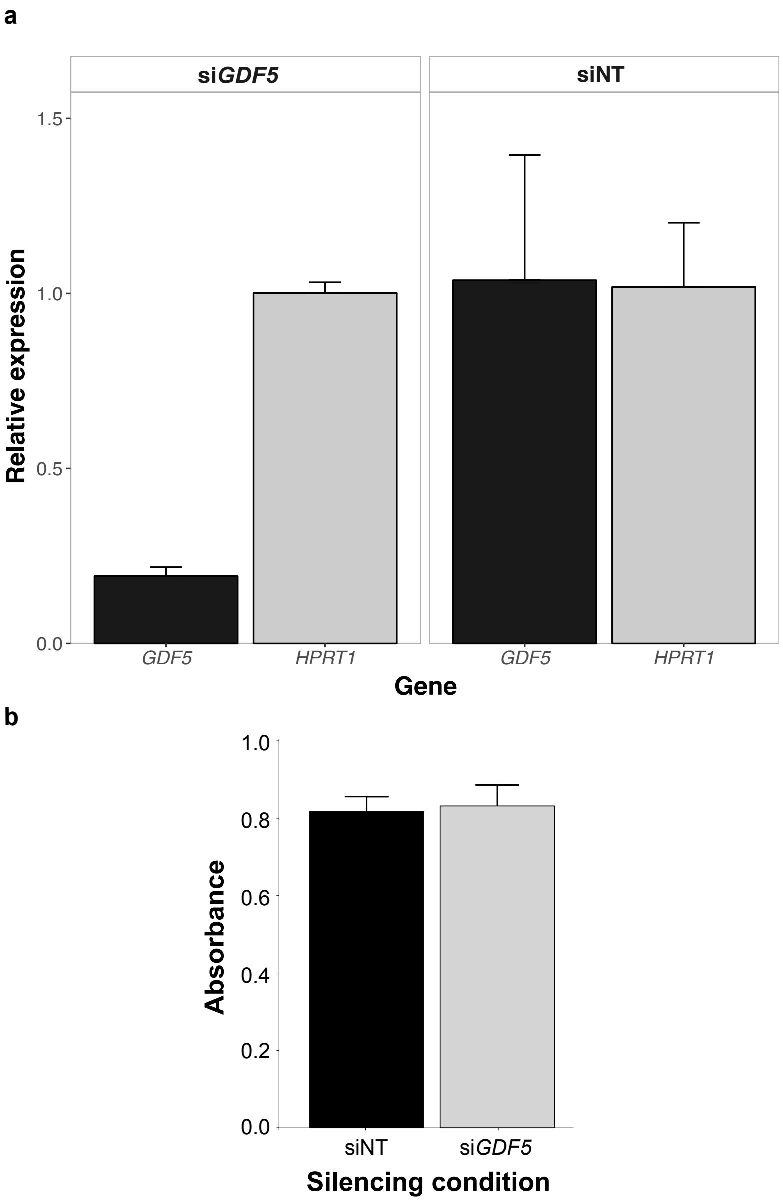

Supplementary Figure 2. RNAi successfully silences *GDF5* and does not cause cell death. a *GDF5* was silenced with siRNA and >75% silencing efficiency was achieved. Expression levels of *GDF5* as well as those of the housekeeping gene, *HPRT1*, were measured. Expression of *GDF5* was (1) specific to *GDF5* and silencing this gene did not affect expression of *HPRT1* (si*GDF5*), and (2) not affected by non-targeted silencing (siNT). **b** HCM cell absorbance was measured before treating the cells with doxorubicin and cell viability was not affected by si*GDF5* alone.

Supplementary Tables

Supplementary Table 1. Differential gene expression of TWAS associated genes in each tissue

	GDF5		*FRS2*		*HDDC2*		*EEF1B2*	
**Tissue**	**Z-score**	***P***	**Z-score**	***P***	**Z-score**	***P***	**Z -score**	***P***
Adipose-Subcutaneous	-4.30	1.70×10^{-5}			3.53	4.15×10^{-4}		
Adipose-Visceral (Omentum)					1.63	0.10		
Adrenal Gland					0.16	0.87		
Artery-Aorta					2.55	0.01		
Artery-Coronary					3.32	9.01×10^{-4}		
Artery-Tibial			-1.07	0.29	3.14	1.70×10^{-3}		
Brain-Anterior cingulate cortex (BA24)					1.51	0.13		
Brain-Cerebellum					-2.36	0.02		
Brain-Cortex					4.01	6.08×10^{-5}		
Brain-Frontal Cortex (BA9)					2.30	0.02		
Brain-Putamen (basal ganglia)	-0.59	0.55						
Breast-Mammary Tissue			0.78	0.43	2.97	3.00×10^{-3}		
Cells-EBV-transformed lymphocytes					2.06	0.04		
Cells-Transformed fibroblasts			2.29	0.02	2.63	8.49×10^{-3}		
Colon-Sigmoid	0.11	0.92			3.08	2.05×10^{-3}		
Colon-Transverse			3.61	3.12×10^{-4}	3.44	5.91×10^{-4}		
Esophagus-Gastroesophageal Junction	-1.54	0.12	1.08	0.28	3.28	1.03×10^{-3}		
Esophagus-Mucosa					3.32	9.01×10^{-4}		
Esophagus-Muscularis	-1.19	0.23	0.12	0.91	3.34	8.38×10^{-4}	-0.51	0.61
Heart-Atrial Appendage	-1.53	0.13			3.61	3.04×10^{-4}		
Heart-Left Ventricle			-0.96	0.34	3.30	9.79×10^{-4}		

Lung	1.63	0.10			3.44	5.76×10^{-4}		
Minor Salivary Gland					2.99	2.79×10^{-3}		
Muscle-Skeletal			-1.24	0.21	2.85	4.40×10^{-3}	-1.57	0.12
Nerve-Tibial			2.30	0.02				
Ovary					2.19	0.03		
Pancreas			4.07	4.67×10^{-5}	3.13	1.75×10^{-3}		
Pituitary	-1.89	0.06			1.32	0.19		
Skin-Not Sun Exposed (Suprapubic)					3.35	8.11×10^{-4}		
Skin-Sun Exposed (Lower leg)			1.39	0.17	2.77	5.63×10^{-3}		
Small Intestine-Terminal Ileum					3.07	2.17×10^{-3}		
Spleen			2.39	0.02	-0.56	0.58		
Stomach			-1.99	0.05	3.51	4.51×10^{-4}		
Testis	1.47	0.14			-1.77	0.08		
Thyroid	0.87	0.39	1.90	0.06	3.26	1.11×10^{-3}		
Vagina							-3.97	7.24×10^{-5}
Whole Blood			-0.19	0.85	2.76	5.72×10^{-3}		

Shaded cells indicate no differential expression

Supplementary Table 2. Publicly-available and cardiotoxic/cardioprotective gene sets

Gene Set	Description	Number of genes	Reference(s)
**Publicly-available gene sets**			
FDA-approved drug targets	Genes whose protein products are targets for FDA-approved drugs	385	7–11
Drug targets (Nelson et al., 2012)	Drug targets	201	12,13
All dominant genes	OMIM disease genes that follow autosomal dominant inheritance	709	14,15
All recessive genes	OMIM disease genes that follow autosomal recessive inheritance	1183	14,15
Essential in culture	Genes essential in human cell lines	283	16
Essential in mice	Genes intolerant to homozygous knockout in mice	2454	17–19
Genes nearest to GWAS peaks	Genes closest to GWAS hits (p<5E-8) in the NHGRI GWAS catalog	6336	20

DNA repair genes (Wood et al., 2005)	Human DNA repair genes	178	21, Updated table: https://www.mdanderson.org/documents/Labs/Wood-Laboratory/human-dna-repair-genes.html
DNA repair genes (Kang et al., 2012)	DNA repair genes derived from DNA repair pathways	151	22
ClinGen haploinsufficient genes	Genes with evidence for dosage pathogenicity according to the ClinGen Dosage Sensitivity Map	294	23
Olfactory receptors	Olfactory receptors	371	24
Genes with any disease association reported in ClinVar	All genes in ClinVar for which there is at least one pathogenic or likely pathogenic variant	3078	25
Kinases	Uniprot's list of protein kinases	347	26–29
GPCRs	GPCR list from guidetopharmacology.org and from UniProt	759	29–31
Natural product targets	Targets of natural products	37	32
BROCA - Cancer Risk Panel	Cancer risk panel consisting of genes involved in various cancers for individuals	66	http://tests.labmed.washington.edu/BROCA

	suspected to have a hereditary cancer predisposition		
ACMG V2.0	List of genes to be reported as incidental or secondary findings developed by the ACMG	59	33
GPI-anchored proteins	Proteins in UniProt that are GPI-anchored	135	29
Universe	Protein-coding genes according to HGNC	19 194	34
LoF intolerant	Genes deemed intolerant to loss of function mutations in ExAC	3230	35
**Cardiotoxic/cardioprotective gene sets**			
Cluster 1	Genes down-regulated in hiPSC-CMs across a gradient of 5 different concentrations of doxorubicin	3062	36
Cluster 2	Genes initially up-regulated and then further down-regulated in hiPSC-CMs across a gradient of 5 different concentrations of doxorubicin	3517	36

Cluster 3	Genes up-regulated in hiPSC-CMs across a gradient of 5 different concentrations of doxorubicin	2026	36
Cluster 4	Genes down-regulated at lower concentrations and up-regulated at higher concentrations across a gradient of 5 different concentrations of doxorubicin in hiPSC-CMs	1150	36
Cluster 5	Genes up-regulated at lower concentrations and down-regulated at higher concentrations across a gradient of 5 different concentrations of doxorubicin in hiPSC-CMs	1419	36
Cluster 6	Genes down-regulated at low concentrations but then partially recover at higher concentrations across a gradient of 5 different concentrations	1143	36

	of doxorubicin in hiPSC-CMs		
ATRA up	Genes up-regulated in response to treating H9c2 cells with ATRA	76	N/A
ATRA down	Genes down-regulated in response to treating H9c2 cells with ATRA	175	N/A
ATRA	All genes up- or down-regulated in response to treating H9c2 cells with ATRA	251	N/A

Supplementary Table 3. Significantly enriched gene sets in heart and arterial tissues

	Heart and arterial tissues			Heart tissue		
Gene set	Mean Z^2 (gene set)	Mean Z^2 (all genes)	P^a	Mean Z^2 (gene set)	Mean Z^2 (all genes)	P^a
**Publicly-available gene sets**						
Essential in mice	0.91	0.83	0.16	0.95	0.84	0.07
Essential in culture	1.09	0.84	0.04	1.09	0.84	0.12
Human LoF intolerant	0.92	0.83	0.06	0.95	0.84	1.00
**Cardiotoxic/cardioprotective gene sets**						
ATRA (downregulated)	0.98	0.84	0.41	1.15	0.85	0.61
Cluster 5[b]	0.85	0.84	1.00	0.90	0.85	0.48

[a]Bonferroni adjusted

[b]Described by Knowles *et al.*[36]

LoF indicates loss of function and ATRA indicates all-trans retinoic acid.

Supplementary Table 4. Comparison of discovery and replication studies

	Aminkeng *et al.*	Schneider *et al.*
Sample size	280 (32 cases, 248 controls)	845 (51 cases, 794 controls)
Population	Children	Adults
Ancestry	European	European
Major cancer type	Acute lymphoblastic leukemia	Breast
GWAS covariates	• Age at start of treatment<br>• Cumulative anthracycline dose<br>• Tumour type (acute lymphoblastic leukemia, Ewing's sarcoma, rhabdomyosarcoma)<br>• Cardiac radiation therapy	• Age<br>• Menopausal status<br>• Experimental arm<br>• Tumour grade<br>• Body surface area<br>• Hypertension during therapy<br>• Use of antihypertension medications at baseline or antihypertensive therapy added during treatment

Supplementary References

1. Greene RF, Collins JM, Jenkins JF, Speyer JL, Myers CE. Plasma Pharmacokinetics of Adriamycin and Adriamycinol: Implications for the Design of in Vitro Experiments and Treatment Protocols. *Cancer Res*. 1983;43(7):3417-3421.

2. Liu J, Zheng H, Tang M, Ryu Y-C, Wang X. A therapeutic dose of doxorubicin activates ubiquitin-proteasome system-mediated proteolysis by acting on both the ubiquitination apparatus and proteasome. *Am J Physiol-Heart Circ Physiol*. 2008;295(6):H2541-H2550. doi:10.1152/ajpheart.01052.2008

3. Wickham H. *Ggplot2: Elegant Graphics for Data Analysis*. Springer-Verlag; 2009. Accessed November 13, 2018. //www.springer.com/gp/book/9780387981413

4. Kassambara, Alboukadel. ggpubr: “ggplot2” based publication ready plots. R package version 0.2.2.

5. Bray NL, Pimentel H, Melsted P, Pachter L. Near-optimal probabilistic RNA-seq quantification. *Nat Biotechnol*. 2016;34(5):525-527. doi:10.1038/nbt.3519

6. Pimentel H, Bray NL, Puente S, Melsted P, Pachter L. Differential analysis of RNA-seq incorporating quantification uncertainty. *Nat Methods*. 2017;14(7):687-690. doi:10.1038/nmeth.4324

7. Knox C, Law V, Jewison T, et al. DrugBank 3.0: a comprehensive resource for ‘Omics’ research on drugs. *Nucleic Acids Res*. 2011;39(suppl_1):D1035-D1041. doi:10.1093/nar/gkq1126

8. Law V, Knox C, Djoumbou Y, et al. DrugBank 4.0: shedding new light on drug metabolism. *Nucleic Acids Res*. 2014;42(D1):D1091-D1097. doi:10.1093/nar/gkt1068

9. Wishart DS, Knox C, Guo AC, et al. DrugBank: a comprehensive resource for in silico drug discovery and exploration. *Nucleic Acids Res*. 2006;34(suppl_1):D668-D672. doi:10.1093/nar/gkj067

10. Wishart DS, Knox C, Guo AC, et al. DrugBank: a knowledgebase for drugs, drug actions and drug targets. *Nucleic Acids Res*. 2008;36(suppl_1):D901-D906. doi:10.1093/nar/gkm958

11. Wishart DS, Feunang YD, Guo AC, et al. DrugBank 5.0: a major update to the DrugBank database for 2018. *Nucleic Acids Res*. 2018;46(D1):D1074-D1082. doi:10.1093/nar/gkx1037

12. Nelson MR, Wegmann D, Ehm MG, et al. An Abundance of Rare Functional Variants in 202 Drug Target Genes Sequenced in 14,002 People. *Science*. 2012;337(6090):100-104. doi:10.1126/science.1217876

13. Russ AP, Lampel S. The druggable genome: an update. *Drug Discov Today*. 2005;10(23):1607-1610. doi:10.1016/S1359-6446(05)03666-4

14. Berg JS, Adams M, Nassar N, et al. An informatics approach to analyzing the incidentalome. *Genet Med*. 2013;15(1):36-44. doi:10.1038/gim.2012.112

15. Blekhman R, Man O, Herrmann L, et al. Natural Selection on Genes that Underlie Human Disease Susceptibility. *Curr Biol*. 2008;18(12):883-889. doi:10.1016/j.cub.2008.04.074

16. Hart T, Brown KR, Sircoulomb F, Rottapel R, Moffat J. Measuring error rates in genomic perturbation screens: gold standards for human functional genomics. *Mol Syst Biol*. 2014;10(7):733. doi:10.15252/msb.20145216

17. Blake JA, Bult CJ, Kadin JA, Richardson JE, Eppig JT. The Mouse Genome Database (MGD): premier model organism resource for mammalian genomics and genetics. *Nucleic Acids Res*. 2011;39(suppl_1):D842-D848. doi:10.1093/nar/gkq1008

18. Georgi B, Voight BF, Bućan M. From Mouse to Human: Evolutionary Genomics Analysis of Human Orthologs of Essential Genes. *PLOS Genet*. 2013;9(5):e1003484. doi:10.1371/journal.pgen.1003484

19. Liu X, Jian X, Boerwinkle E. dbNSFP v2.0: A Database of Human Non-synonymous SNVs and Their Functional Predictions and Annotations. *Hum Mutat*. 2013;34(9):E2393-E2402. doi:10.1002/humu.22376

20. MacArthur J, Bowler E, Cerezo M, et al. The new NHGRI-EBI Catalog of published genome-wide association studies (GWAS Catalog). *Nucleic Acids Res*. 2017;45(D1):D896-D901. doi:10.1093/nar/gkw1133

21. Wood RD, Mitchell M, Lindahl T. Human DNA repair genes, 2005. *Mutat Res Mol Mech Mutagen*. 2005;577(1):275-283. doi:10.1016/j.mrfmmm.2005.03.007

22. Kang J, D'Andrea AD, Kozono D. A DNA Repair Pathway–Focused Score for Prediction of Outcomes in Ovarian Cancer Treated With Platinum-Based Chemotherapy. *JNCI J Natl Cancer Inst*. 2012;104(9):670-681. doi:10.1093/jnci/djs177

23. Rehm HL, Berg JS, Brooks LD, et al. ClinGen — The Clinical Genome Resource. *N Engl J Med*. 2015;372(23):2235-2242. doi:10.1056/NEJMsr1406261

24. Mainland JD, Li YR, Zhou T, Liu WLL, Matsunami H. Human olfactory receptor responses to odorants. *Sci Data*. 2015;2:150002. doi:10.1038/sdata.2015.2

25. Landrum MJ, Lee JM, Riley GR, et al. ClinVar: public archive of relationships among sequence variation and human phenotype. *Nucleic Acids Res*. 2014;42(D1):D980-D985. doi:10.1093/nar/gkt1113

26. Hunter T. Signaling—2000 and Beyond. *Cell*. 2000;100(1):113-127. doi:10.1016/S0092-8674(00)81688-8

27. Manning G, Whyte DB, Martinez R, Hunter T, Sudarsanam S. The Protein Kinase Complement of the Human Genome. *Science*. 2002;298(5600):1912-1934. doi:10.1126/science.1075762

28. Miranda-Saavedra D, Barton GJ. Classification and functional annotation of eukaryotic protein kinases. *Proteins Struct Funct Bioinforma*. 2007;68(4):893-914. doi:10.1002/prot.21444

29. UniProt Consortium T. UniProt: the universal protein knowledgebase. *Nucleic Acids Res*. 2018;46(5):2699-2699. doi:10.1093/nar/gky092

30. Alexander SP, Christopoulos A, Davenport AP, et al. THE CONCISE GUIDE TO PHARMACOLOGY 2017/18: G protein-coupled receptors: THE CONCISE GUIDE TO PHARMACOLOGY 2017/18: G protein-coupled receptors. *Br J Pharmacol*. 2017;174:S17-S129. doi:10.1111/bph.13878

31. Harding SD, Sharman JL, Faccenda E, et al. The IUPHAR/BPS Guide to PHARMACOLOGY in 2018: updates and expansion to encompass the new guide to IMMUNOPHARMACOLOGY. *Nucleic Acids Res*. 2018;46(D1):D1091-D1106. doi:10.1093/nar/gkx1121

32. Dančík V, Seiler KP, Young DW, Schreiber SL, Clemons PA. Distinct Biological Network Properties between the Targets of Natural Products and Disease Genes. *J Am Chem Soc*. 2010;132(27):9259-9261. doi:10.1021/ja102798t

33. Kalia SS, Adelman K, Bale SJ, et al. Recommendations for reporting of secondary findings in clinical exome and genome sequencing, 2016 update (ACMG SF v2.0): a policy statement of the American College of Medical Genetics and Genomics. *Genet Med*. 2017;19(2):249-255. doi:10.1038/gim.2016.190

34. Gray KA, Yates B, Seal RL, Wright MW, Bruford EA. Genenames.org: the HGNC resources in 2015. *Nucleic Acids Res*. 2015;43(Database issue):D1079-D1085. doi:10.1093/nar/gku1071

35. Lek M, Karczewski KJ, Minikel EV, et al. Analysis of protein-coding genetic variation in 60,706 humans. *Nature*. 2016;536(7616):285-291. doi:10.1038/nature19057

36. Knowles DA, Burrows CK, Blischak JD, et al. Determining the genetic basis of anthracycline-cardiotoxicity by molecular response QTL mapping in induced cardiomyocytes. *eLife*. 2018;7:e33480.
